# Young People’s Experiences Using a Digital Mental Health Tool to Support Their Care in a Real-World Service: Lived Experience–Led Qualitative Study

**DOI:** 10.2196/70154

**Published:** 2025-06-23

**Authors:** Carla Gorban, Min K Chong, Adam Poulsen, Ashlee Turner, Haley M LaMonica, Sarah McKenna, Elizabeth M Scott, Ian B Hickie, Frank Iorfino

**Affiliations:** 1 Brain and Mind Centre, University of Sydney, 94 Mallett Street, Camperdown, 2050, Australia, 61 9351 0584

**Keywords:** youth mental health, digital navigator, digital mental health, measurement-based care, digital mental health tools, health services research

## Abstract

**Background:**

The uptake of digital mental health tools (DMHTs) in mental health services is suboptimal, limiting key avenues to facilitate personalized and measurement-based care. This misses critical opportunities for enhanced patient-clinician communication, improved assessment, and early intervention.

**Objective:**

This paper aims to understand young people’s experiences and perceptions of engaging with a DMHT to support their care in a real-world setting over time.

**Methods:**

This study is part of a larger randomized controlled trial, where an added human support, the digital navigator (DN), provided technological and engagement assistance for young people to use a DMHT as part of usual care. The DN conducted 118 semistructured interviews with 73 young people (mean age 22.7, SD 2.7 y) at baseline and 3-, 6-, and 12-month follow-up visits.

**Results:**

We found that the majority of young people were enthusiastic about incorporating a DMHT into their care when they understood its potential to facilitate shared decision-making and enhance self-awareness of their mental health. Notably, the DN’s support was effective in fostering this understanding at the initial stages of implementation. However, it was evident that the lack of clinician buy-in for using the DMHT posed a risk of disillusionment to young people’s sustained engagement with the tool. Young people perceived that clinician uptake of the tool was poor, limiting its perceived value addition and sustainability.

**Conclusions:**

Young people want to use DMHTs in their care and DNs can effectively facilitate implementation through ongoing engagement and technical support. However, successful implementation of DMHTs also depends on broader systemic factors, particularly on clinician and service engagement. Future research should examine how to address these contextual barriers and optimize DN support for implementation and sustained engagement of DMHTs.

## Introduction

Youth mental health has emerged as a global crisis [1], with mental disorders affecting more than 1 in 10 individuals aged between 5 and 24 years and accounting for the highest disease burden in this age group [2]. Despite the widespread use of psychotherapies and pharmacotherapies, their overall effectiveness remains modest [3,4], highlighting the need to enhance personalization and precision in treatment [5].

Measurement-based care (MBC) offers a promising solution by systematically collecting standard measurements to inform and guide mental health care. It involves 2 main components, routine collection and review of client-reported outcomes, to monitor progress, evaluate treatment plans, and inform shared decision-making [6]. Digital mental health tools (DMHTs) have been introduced in services to facilitate MBC, increasing accessibility to multidimensional measures and assisting with data interpretation by visualizing symptom progress [7,8]. Such DMHTs are able to detect at-risk clients [9,10], support client-clinician communication [11], and improve treatment engagement and outcomes [12-14]. Given the challenges associated with accurately predicting clients who are more likely to deteriorate in care [15], the iterative process of symptom tracking and review using DMHTs ensures that treatment is data-informed [16] and personalized to individuals’ changing needs [17]. This is particularly beneficial for young people who often present with complex and heterogeneous symptoms that do not fit into specific diagnostic criteria [18,19].

Despite its potential, implementation of DMHTs in mental health services remains challenging. Meaningful engagement and complete integration of DMHTs into services are crucial factors for success [20-22]. Yet, barriers to client, clinician, and organizational uptake are complex. These challenges are closely tied to various aspects of service capacity: high service demand can limit the time for adoption, insufficient education and training reduces clinician confidence in effectively using these tools, and a lack of leadership support undermines organizational commitment to integrating DMHTs into routine care [23-25]. Furthermore, conflicting reports exist on the feasibility and acceptability of DMHTs. For example, while some clinicians perceive regular monitoring as overly time-consuming for their clients [26,27], a meta-analysis demonstrates high adherence and acceptability rates even among people with severe mental illnesses [28]. This discrepancy, along with the suboptimal uptake of DMHTs [29,30], indicate a need for a deeper understanding of the factors that influence young people’s experience of and engagement with these tools, especially over time.

A potential solution to address these barriers is the introduction of a digital navigator (DN) role into health services [31]. A DN can optimize the value of DMHTs to clinical teams by being the conduit between the client, the clinician, and services. By providing technical and engagement support, they facilitate the implementation and adoption of these digital innovations in clinical settings [31,32]. They are responsible for improving accessibility, helping services to integrate new tools into clinical governance, and enhancing client engagement through regular support [33,34]. Furthermore, a DN with lived experience can provide an added benefit of peer support, fostering trust and accountability for clients using DMHTs in care [35].

Therefore, this study aims to assess young people’s experiences and real-time changing perceptions of integrating a DMHT (Innowell, Innowell Pty Ltd), designed to facilitate highly personalized, measurement-based care and symptom data review into their routine care, with the support from a DN. The DN conducted semistructured interviews at 4 time points (baseline and 3-, 6-, and 12-month follow-ups) to understand their evolving engagement patterns and perceived value-add of the DMHT in a real-world clinical setting over time.

## Methods

### Study Context

This study is part of a larger, multisite, 24-month randomized controlled trial examining the clinical effectiveness of highly personalized MBC for young people (15‐25 y) engaged in mental health services [36]. The ongoing trial is being conducted at the University of Sydney’s Brain and Mind Centre (Australia) and affiliated youth centers that treat young people with mental illnesses. While the broader trial involves multiple sites, the qualitative data presented in this paper were collected from a single site: Mind Plasticity, a private, specialist consortia based in inner-city Sydney that provides multidisciplinary care to individuals requiring mental health support. Participant recruitment, screening for eligibility, and obtaining informed consent are facilitated by the trial research team, per the trial protocol published elsewhere [36].

Both treatment arms have access to the DMHT—Innowell—and receive ongoing support from a DN. Innowell is a DMHT that seeks to promote better mental health outcomes by facilitating access to comprehensive multidimensional assessment and real-time results to inform clinical care [37]. The DN role focuses on providing peer support to participants, aiding their understanding of how to use the tool in their care. This includes motivating them to use Innowell, developing strategies to enhance their engagement, resolving technical issues experienced by participants, carers, and clinicians, and performing data collection.

### The Digital Navigator

The DN (author CG) was embedded in the service as part of the trial design. The DN’s previous professional experience in a public youth mental health service, where she was involved in a service redesign that resulted in the implementation of Innowell, was instrumental to her approach to the DN role. Lived experience is a key factor of this role and this expertise was crucial to forming rapport with participants to share information about their current care and how Innowell can enhance it [38]. As the DN had previous professional experience with Innowell, her training was more practical as part of a pre-trial phase, which focused on optimizing the role for the specific service [32].

Within the trial, the DN’s duties were to regularly interact with all participants, while tracking Innowell engagement data only for those in the intervention arm. The DN’s focus was to monitor the frequency of Innowell use among the intervention arm participants, to ensure their consistent symptom tracking. When the DN noticed that a participant had not entered symptom data for more than 21 days, this was marked as a risk of disengagement. Subsequently, this prompted the DN to contact the participant to address any concerns regarding the use of Innowell. This peer support aimed to encourage participants to resume using Innowell.

### Data Collection

The trial protocol [36] details the full set of data items and collection processes, which includes a structured clinical interview for *Diagnostic and Statistical Manual of Mental Disorders* [39], standardized clinical assessments, and ongoing data collected via Innowell. Relevant data collected and analyzed here includes research observations and field notes recorded by the DN during 4 “study visits” with young people conducted at key time points: baseline and 3-, 6-, and 12-month follow-ups. A final visit will occur at the trial’s end (24 months). Study visits were initiated by a research assessor, that is, a member of the research team who conducted clinical assessments with participants. After that, the DN had a brief follow-up discussion (approximately 5 min) with participants to provide ongoing support and complete data collection.

Textbox 1 details the discussion topics that guided the study visits between the DN and participants, and Multimedia Appendix 1 collates the most common questions asked by the DN. The discussion topics and questions were adapted to ensure a natural conversational flow and account for various other factors, including, for instance, a participant’s current level of care, previous experience of using Innowell in care, and mental health status. Study visits were conducted in-person, over the phone, or via Zoom teleconferencing software (Zoom Communications), and ranged between 5 and 30 minutes. Multiple participation modalities were offered to enable young people to participate via their preferred method to maximize safety, comfort, and scheduling. In-person study visits were conducted at the Brain and Mind Centre to provide participants with a safe, comfortable, and private environment, with a nearby supporting mental health clinician made readily available. Similarly, as an equal member of a participant’s care team, a crucial factor in encouraging engagement with Innowell [32], the DN is well positioned to inspire a safe and comfortable environment, open feedback, and familiarity among participants as a data collector. All semistructured interviews were conducted with risk protocols in place for the DN, ensuring clear boundaries between the DN’s peer support role and clinical responsibilities, and escalation protocols when required. These were established as part of the trial to ensure the safety of both the DN and the participants.

**Textbox 1. Discussion topic guide**

- Your Innowell use.
- Introduction to personalized and measurement-based care (MBC) model.
- Clinician use.
- Current care.
- Improving accessibility.

Data collected included research observations (eg, participant behavior and environmental factors during interviews) and field notes (eg, interviewer reflections and interpretations during interviews). Interviews were not recorded and transcribed, instead the research observations and field notes were combined to form the interview data, which documents views on how the DN role functions (from the participant’s and DN’s perspective); participant experiences with their care and Innowell (eg, Innowell onboarding completion and duration of use); contextual information (eg, participant demographics, trial enrolment status and time point, changes in participant’s care delivery and life circumstances, regularity of clinical appointments, etc); and verbatim responses to questions during the discussion. Field notes and research observations enable researchers to collect rich contextual information about the participants’ lives and study engagement [40], and thus are suitable for constructing rich descriptions of the study visits, participant responses, and researcher reflections as data for thematic analysis [41,42]. Interview data was later deidentified, collated, and organized by individual participants in question-response format for data analysis.

### Data Analysis

This study used reflexive thematic analysis to develop and report themes based on patterns of shared meaning in the interview data [43,44]. Established thematic techniques reported by Braun and Clarke [45] guided this study, informing 6 iterative, flexible phases: familiarizing yourself with the data, generating initial codes, searching for themes, reviewing potential themes, defining and naming themes, and producing the report. The data was interpreted using an inductive approach. NVivo 14 (Lumivero) was used to collate and code interview data.

Here, the following thematic analysis processes were completed. First, 3 co-authors (CG, MKC, and AP) independently reviewed 5 interviews and generated initial codes. Following thematic analysis best practice, generating codes involved the 3 co-authors “closely reading the data, considering what is interesting and important, and then assigning a code label that captures your understanding of the meaning of a segment of the data” [46]. Codes were not predetermined before analysis, were subject to evolve as understanding deepens, and were labeled with descriptions of why some data are relevant to the research question [46]. Following that, in discussion, the initial codes were reviewed, combined, and revised, and new codes were developed. Importantly, noting an adaption of the reflexive thematic analysis method here [47], a mutable coding framework was created collaboratively to be used as a flexible guide for the remaining analysis to ensure that the multiple co-authors could independently code using a shared language. After that, using the flexible coding framework, 4 co-authors (CG, MKC, AP, and AT) each independently reviewed and coded a part of the total remaining interview data. After that, in group discussion, variability between coauthors’ codes was resolved to ensure that codes were mutually agreed upon, and the final set of codes were consistently applied across the combined dataset. Then, on 2 occasions, the 4 coauthors (CG, MKC, AP, and AT) met to iteratively develop, review, and label the themes in consultation with the senior author (FI). Multimedia Appendix 2 shows an in-depth description of how specific themes were finalized. Through this collaborative and iterative process, consensus was reached on the final set of codes and themes, ensuring coherence and consistency.

Following best practices for thematic analysis, the principle of information power is preferred to data saturation in determining whether data provides a sufficient basis for the proposed analysis [46]. As per the information power principle, a study needs fewer participants when the study primarily addresses a narrow aim (ie, young people’s experiences and perceptions of engaging with Innowell), the participants represent similar experiences and knowledge (ie, young people), the study is grounded in existing theory (ie, MBC), an experienced interviewer conducts it, and it addresses a single case analysis [48]. The sample size here adequately satisfies the typically expected 15‐30 participants range for single case studies [48], and thus, the study holds ample information power to develop new knowledge.

### Ethical Considerations

This clinical trial was approved by the Human Research Ethics Committee of the Sydney Local Health District (HREC X22-0042 and 2022/ETH00725)" and all participants gave informed consent for their qualitative data to be used for research purpose. All data had been de-identified. Participants received a AUD$50 Coles/Myer gift voucher at the completion of the baseline assessment and a further AUD$50 voucher at the completion of the 12th assessment.

## Results

### Participants

A total of 73 young people aged between 15 and 25 years participated in this study. A total of 118 interviews were conducted: 73 at baseline, 5 at 3-month, 29 at 6-month, and 2 at 12-month follow-up visits. The mean age of participants was 22.7 (SD 2.7) years and 51 (69.9%) participants were female. Table 1 shows the mental health diagnoses of participants.

**Table 1.** Demographic and clinical presentation of participants.

Lifetime diagnosis of mental disorders	Participants (N=73), n (%)
Major depressive disorder	53 (72.6)
Anxiety disorder	60 (82.2)
Bipolar I disorder	4 (5.5)
Bipolar II disorder	7 (9.6)
Substance use disorder	1 (1.4)

### Thematic Analysis

A total of 4 themes and 6 subthemes were developed based on patterns of shared meaning in the data using reflexive thematic analysis (see Table 2). The themes (and subthemes) include: “DMHTs are beneficial for communication and collaboration” (subthemes: promoting collaborative care and easing communication), “Data is an important tool in care” (subthemes: representing mental health conditions through accurate data and enhancing self-awareness with longitudinal data), “The impact of the DN in optimizing engagement and value” (subthemes: inspiring engagement and creating understanding), and “Promoting value for myself and others.”

**Table 2.** Theme names and descriptions.

Theme name	Theme description
DMHTs^a^ are beneficial for communication and collaboration	Relationships and communication in care and the introduced role of DMHTs.
Data is an important tool in care	How data is impactful and used by young people and clinicians in care.
The impact of the DN^b^ in optimizing engagement and value	Key roles of the DN for improving understanding and engagement of DMHTs and to advance toward personalization.
Promoting value for myself and others	Meaningful ways DMHTs can be used to add value to oneself and for the broader community via informing service provision change.

a DMHT: digital mental health tool.

b DN: digital navigator.

**Figure 1.**
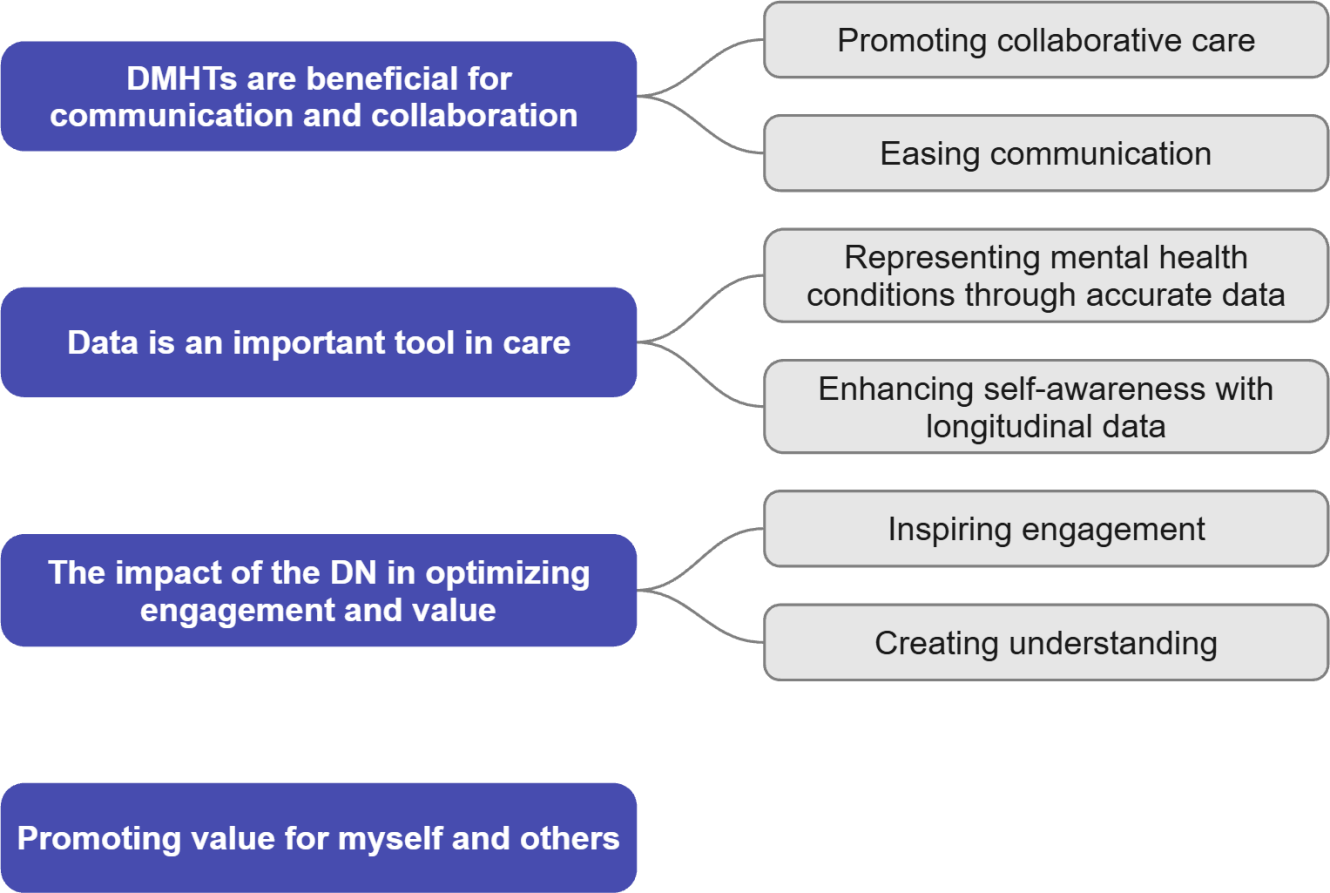
Final thematic map. DMHT: digital mental health tool; DN: digital navigator.

Figure 1 visualizes the final thematic map, indicating the 4 themes developed during analysis. Per best practices for reporting thematic analysis [45], Multimedia Appendix 2 shows the complete process of thematic development.

### DMHTs Are Beneficial for Communication and Collaboration

#### Promoting Collaborative Care

Participants experienced a positive change in the quality of their care when their multidimensional assessment data was collaboratively discussed with clinicians. They found that the DMHT, Innowell, effectively coordinated care between various health professionals (eg, general practitioner, psychologist, and psychiatrist), tracked medication response, and informed care plans based on individual progress. The collaborative use of collected symptom data with clinicians heavily influenced participants motivation, engagement, and ability to have meaningful experiences with Innowell.

Notably, participants’ perceived value of Innowell increased when their data was used by clinicians to inform and modify treatment plans in response to their changing needs. For example, a participant found Innowell “most helpful” when it allowed them to “bring it up with [psychiatrist] and use it as a tool to review data together”. They knew “from Innowell that this is how [they’ve] been and being able to pull it up and show [their psychiatrist]” led them to go into “immediate damage control” (Participant 1, 3-month visit). Similarly, participants liked that collaboratively reviewing their data with clinicians could facilitate proactive care. Before using Innowell, they were hopeful that regular symptom data input and review will allow clinicians to detect changes in their mental health early, so that they could be “addressed now, rather than later when it’s too late or missed it” (Participant 2, baseline visit). Multimedia Appendix 3 shows additional quotes that illustrate participants’ emphasis on the impact of the collaborative nature of Innowell.

However, most participants expressed that their clinicians did not deeply incorporate Innowell in their care. At baseline visits, most participants were enthusiastic to use Innowell as they learnt about its potential to enhance collaborative care with their clinicians. However, their initial motivation quickly diminished when neither Innowell nor their data was acknowledged or discussed with clinicians during their therapy sessions. The following feedback from a participant at 2 study time points illustrates this view:

*I want to talk more about myself, share my emotions more during the therapy. I feel Innowell will help me communicate this to [my psychiatrist].*

[Participant 3, baseline visit]

*[My psychiatrist] never talked about it [Innowell] but I'm just doing it because of this trial… but it’s not that helpful. Talking with a real person, like now, would be inherently better. It’s just a website now, so I'm just inputting the data, like on auto-mode*.

[Participant 3, 6-month visit]

In addition, participants wanted greater initiative from clinicians to drive collaborative care. At the 12-month follow-up, 1 participant recalled:

*If I turned up at a session and (my clinician) was like “You didn’t use Innowell, we have to do it now to get more out of your appointment,” that would be more motivating for me. It really needs to be used with your clinician*.

[Participant 9, 12-month visit]

Participants expected the use of Innowell to be also “valued by clinician[s].” However, when this expectation was not met during care, a participant expressed feeling “cheated” (Participant 43, 6-month visit) by their clinicians.

#### Easing Communication

Innowell was an effective communication tool for participants who carried guilt and shame around their mental health. Participants usually found it challenging to overcome these emotions and openly communicate their deteriorating mental health to their clinicians. During these periods, showing their symptom data on Innowell made “it a lot easier to communicate how [they were] feeling. [It was] really helpful for [them] to tell the truth about [their] emotions” and it was “easier to answer the questionnaires without feeling the guilt and shame to bring it up to [clinicians]” (Participant 4, 12-month visit).

In addition, the symptom data collected between sessions helped participants to effectively communicate important mental health changes during their appointments. Having data that illustrates their changes in mental health gave them confidence. A participant who used Innowell consistently for few months said that it allowed them to “actually [be] able to voice, and [feel] confident to voice [symptom changes to clinician] because all the information was there” (Participant 1, 3-month visit). They felt that Innowell helped them to feel more prepared for appointments because their data provided “a point of reference for [psychiatrist] but also a very good point of reference for [themselves].*”* (Participant 5, 6-month visit). They reported that this sense of readiness and made the sessions more efficient.

### Data Is an Important Tool in Care

#### Representing Mental Health Conditions Through Accurate Data

Participants expressed different and sometimes contrasting perspectives on the value of standardized assessments for tracking treatment progress. Some participants expressed that they could “trust the answers more because [they knew] it was legitimate” (Participant 6, 3-month visit). Furthermore, the multidimensional measures allowed participants to break down and “differentiate those components that make up [their] mental health and better identify where that underlying feeling [they are] having lies” (Participant 6, 3-month visit) and helped them to “conceptualize what’s going on for [them]” (Participants 7, 3-month visit).

Some contrasting views about standardized assessments were also reported. Although a quantitative representation of mental health provided insights into their symptom trajectories over time, this was not always useful as “some of those questions feel imprecise [to their diagnosis]” and “[it] doesn’t necessarily capture things as deeply” (Participant 8, 6-month visit). Participants believed that their answers to the assessment were “a lot more of a complicated issue [and the assessment on Innowell] maybe doesn’t go into enough detail” (Participant 5, 6-month visit). The inability to provide the additional context to these assessments made participants question “the accuracy in terms of what the data is looking at” (Participant 8, 6-month visit).

#### Enhancing Self-Awareness With Longitudinal Data

**Figure 2.**
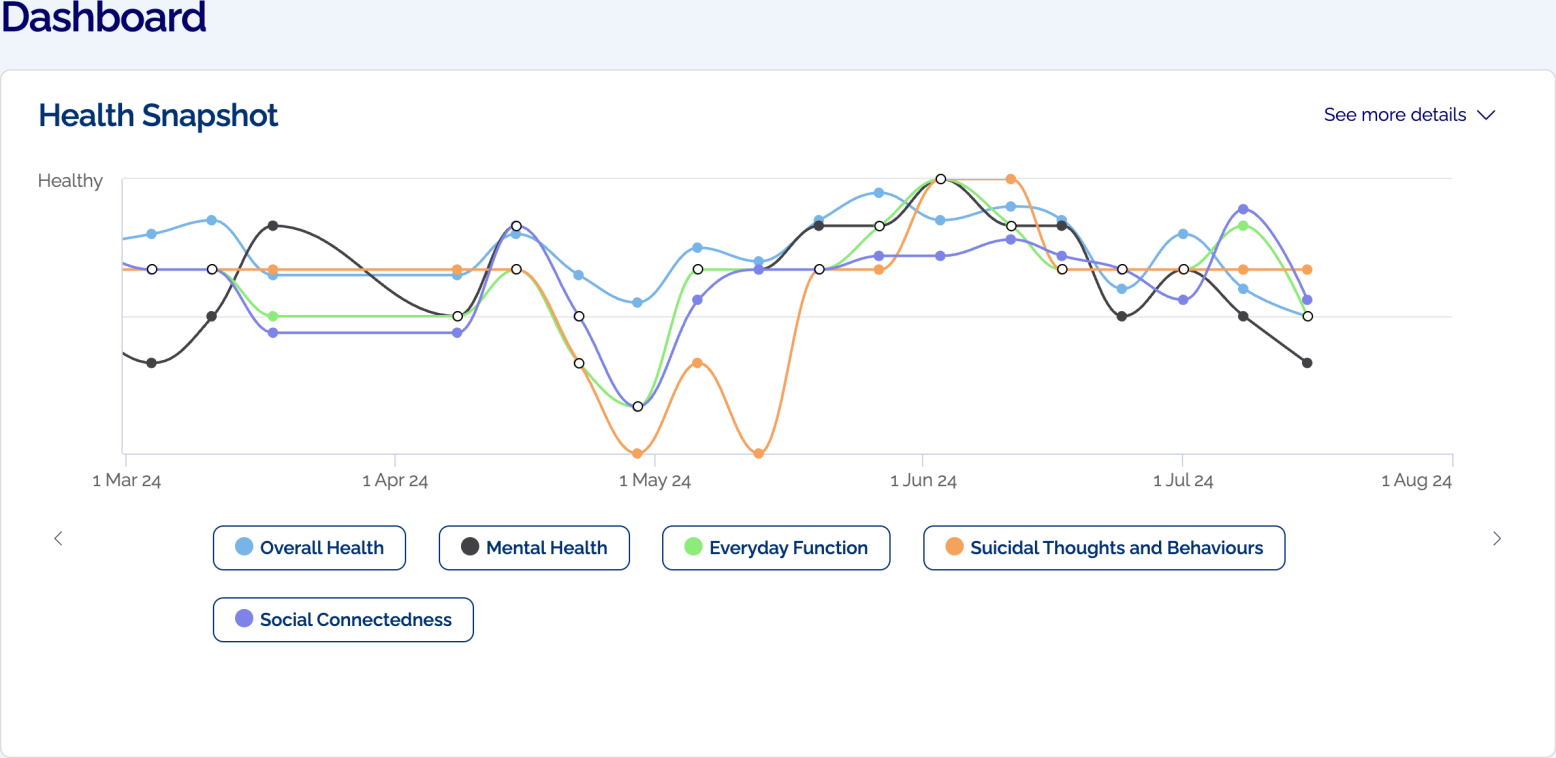
Graph summary of a client’s Innowell.

Participants found the longitudinal symptom data on Innowell to be insightful for self-reflection. In particular, the visual presentation enhanced participants’ understanding of their mental health trajectory over time (see Figure 2). This data-driven self-awareness empowered them to identify “slightest of dips on the graph” and take proactive steps to “help manage [their] symptoms by catching it in time” (Participant 9, 6-month visit).

The insights gained from their personal mental health trajectory served as a motivational factor for some participants. They reported feeling hopeful even during difficult periods of poor mental health because they understood its dynamic nature from previous symptom data. It served as a reminder that positive periods would return. For example, one participant who consistently used Innowell recalled that:

*I’ve never had the opportunity to look back and see when things have been good. In my head, I think you focus on the times you’ve been bad so that becomes all you see in yourself, but it’s nice to look at it [the graphs] and be able to go “hey, I was pretty good there.”*

[Participant 10, 6-month visit]

Furthermore, longitudinal symptom data was used as an extension to their care, particularly for those who had irregular or infrequent appointments (eg, every 3 or 6 months). For these participants, access to Innowell helped them to “keep engaged in [their] mental health” between appointments and meant they “don’t have to check-out completely” (Participant 6, 6-month visit). In addition, using Innowell as a personal record keeping tool was useful when explaining their condition to new health professionals.

### The Impact of the DN in Optimizing Engagement and Value

#### Inspiring Engagement

One of the key responsibilities of the DN within the trial was to routinely encourage participants to complete questionnaires on Innowell to ensure routine outcome monitoring. Participants’ feedback indicated that the DN played a critical role in galvanizing young people to use Innowell from the beginning and sustaining their engagement throughout the trial.

The rapport that formed between participants and the DN through regular contact served as a strong motivator for engagement. The genuine empathy, compassion and lived experience of the DN made participants feel that the DN “actually care[d] about [them] using the technology as part of [their] appointments” (Participant 7, 6-month visit). The importance of human support is exemplified by a participant’s feedback stating that the DN’s personalized text reminders for Innowell completion were “honestly the most helpful” for ongoing use of Innowell and “very useful in terms of helping [them] feel supported to use it” (Participant 11, 6-month visit). In addition, another participant described that it was “a relief to know that a DN is looking at it and monitoring [their] Innowell when updated” and “nice knowing that there’s someone there to support [them] with it if [they] ever need it” (Participant 12, 6-month visit).

Beyond providing engagement support, the DN helped participants to alleviate technical barriers of using Innowell, although the approach and outcome differed case by case. Exemplary approaches revolved around offering in-person support where possible, providing accessibility tips, and supporting creative problem-solving. These are examples from different participants at the 6-month follow-up visits, who initially were not using Innowell regularly due to difficulties with access. These quotes illustrate the added value of the DN in collaborating with young people to find individualized solutions to alleviate barriers and inspire engagement:

*[Innowell] only really works for me when it’s in person…Every time I've been in the clinic, [the DN] has been really good at motivating me to do it…In-person support [from the DN] is absolutely the way to go.*

[Participant 13, 6-month visit]

*[After the DN explained how to access the log in page of Innowell] That’s much better. Now I’ll be able to access it easier. I can see myself doing the questions more consistently now.*

[Participant 14, 6-month visit]

However, there were limitations to alleviating barriers that extended beyond the control of the DN. In most cases, these barriers revolved around clinician engagement and uptake with integrating DMHTs into clinical practice, appointment frequency, duration and type of care, and external technological factors. Multimedia Appendix 3 shows participant quotes that illustrate this.

#### Creating Understanding

For participants, understanding the benefits of Innowell and practical implementation strategies as part of their care were important drivers for ongoing engagement. Informed by their lived experience, the DN had a substantial understanding of participants’ needs and challenges within the mental healthcare system. Therefore, the DN was effective in communicating how participants’ current needs can be addressed through Innowell. Providing explanation on complex concepts such as MBC, self-advocacy and shared decision-making further supported participants’ understanding of the benefits of the tool. Establishing this understanding made the implementation of Innowell “[make] a lot more sense…behind why I should do it and knowing when to do it too” (Participant 15, baseline visit). The tailored suggestions of the DN on how participants could “use Innowell in a way that aligned with my goals for using it in my care” (Participant 1, 6-month visit) were deemed especially helpful.

An exemplary scenario demonstrates the role of the DN in creating understanding of Innowell to motivate engagement. A participant, initially hesitant to use Innowell due to a negative experience at a different mental health service, discussed their concerns with the DN. Recognizing that Innowell had previously been misused with this participant, the DN allayed their reluctance by explaining its purpose and how it could be effectively integrated into their care. The following is a segment from that conversation:

*Explaining the intent of it was really helpful. It [Innowell] was never explained to me how it should be used. It’s really helpful to know not just how I'm meant to use it but also knowing how a clinician is supposed to use it. I can see that when your clinician understands this intent too, it has the potential to be really helpful for the person…I can see how Innowell can work when it’s used properly.*

[*Participant 16, baseline visit*]

### Promoting Value for Myself and Others

Participants often expressed a broad, altruistic perspective on the potential of DMHTs when discussing their expectations and goals for using Innowell as part of their care. Many felt that Innowell could catalyze meaningful and essential transformations across all levels of the mental health care system—from organizational change to empowering themselves and other young people to shape the improvements they seek in their care.

Multiple participants reported to the DN that they were actively seeking ways to improve their current care, explaining that “the therapeutic relationship hasn’t evolved with the needs that [they] have as [they’ve] grown up…even though [they] would like something different” (Participant 17, baseline visit). They even went so far as to say “[The clinician] thinks I’m okay, but actually no, I’m not” (Participant 17, baseline visit). Participants welcomed the integration of DMHTs, seeing it as a way to address perceived limitations in their current care. They felt that “Innowell can help [them] to advocate for what [they] really need” (Participant 17, baseline visit), and it “could make care more well-rounded, more progressive” by “break[ing] down a lot of barriers for young people who receive care” (Participant 18, baseline visit).

However, across different time points, participants voiced concerns about the potential for DMHTs to be implemented poorly by clinicians and services. When Innowell was not incorporated into their sessions, one participant expressed that they would “like the Innowell platform to be seen as part of [their] care. It needs to be seen as an integral component to how clinicians and a service operate.” The participant wanted to “see the platform be more driven and encouraged by clinicians” (Participant 19, 6-month visit). The delay and reluctance of services and clinicians to integrate DMHTs in care was seen as a sign that the healthcare system needs to adapt to young people’s needs and views.

On a broader level, participants viewed their involvement in the trial as an opportunity to create meaningful change in mental healthcare delivery, benefiting future help-seeking young people. They expressed a desire “to be involved in helping develop mental health systems that can better support others” (Participant 15, baseline visit). They believed that “this kind of stuff makes things better for people in the future” (Participant 1, 6-month visit) and that “change will only happen when [their] needs and voices are represented in research, that is linked to empirical evidence to support that change that needs to happen” (Participant 20, baseline visit).

## Discussion

### Principal Findings

This study explores young people’s real-world experiences and evolving engagement with using a DMHT, Innowell, as part of their ongoing care in a real-world, clinical setting in Sydney, Australia. Particularly, the repeated observations and interviews conducted throughout care provided enriched understanding of their evolving perspectives in real time. The findings reveal that young people were enthusiastic about integrating the DMHT in their care when they understood how it can facilitate self-reflection, ease communication burdens, and enhance shared decision-making with their clinicians. The 4 themes developed here demonstrate the value of DMHTs for improving communication and collaboration in care, the usefulness of data as a tool, the significance of promoting its value, and the DN’s role in translating the utility of DMHTs into practical applications. Overall, this study shows that most young people were motivated to adopt the DMHT to improve their mental health care. Notably, although most young people initially expressed interest in integrating the DMHT into their care, their sustained engagement and regular use of Innowell appeared to depend on the level of clinician involvement and its collaborative use in care. Furthermore, the study indicates that young people recognize the crucial role that services play in establishing the infrastructure required for the genuine, holistic adoption of DMHTs, highlighting the importance of proactive service-wide implementation in maximizing their benefits.

### Young People Want to Use Technology

Contrary to views of some mental health professionals [26,49], this study demonstrates that young people are eager to use DMHTs as an integral component of their care. Participants reported that using the data collected from the validated measures in Innowell helped them feel more prepared for therapy sessions, provided an objective overview of their symptoms, and reduced feelings of guilt, shame, and anxiety associated with discussing changes in their mental health with their clinicians. In this way, the DMHT facilitated more open and honest conversations, and therefore, were willing to continue inputting their data. For young people, the primary value-add of using a DMHT was that it gave them an equal voice and enhanced self-advocacy, thereby supporting shared decision-making with clinicians and leading to more efficient, highly-personalized appointments that proactively addressed their real-time changing needs.

Similarly, positive experiences with tracking multidimensional data served as a motivator for sustained engagement with the DMHT. Some young people leveraged their data to gain accurate insights into their mental health trajectories, ultimately inspiring self-driven engagement [50]. A self-reinforcing loop developed for those who consistently used Innowell over a prolonged period: they entered their symptom data, gained insights, and continued tracking due to their positive experiences. Interestingly, these individuals exhibited an inherent drive to continue monitoring their own symptoms by using the DMHT regardless of their clinician’s involvement. However, as observed from the qualitative interviews with the DN, mainly at 6- and 12-month follow-ups, there was a substantial decline in engagement with using Innowell shortly after the participants entered the trial. Therefore, this experience of benefiting from self-tracking their data was only reported among a minority of participants. Many participants disengaged from using Innowell regularly before they could discover the benefits of tracking their data over time. Hence, the study suggests that without these positive experiences during the early stages of using the DMHT in care, its prolonged use is at risk of being limited.

### Collaboration is Key for Meaningful Engagement

A total of 2 interdependent factors were identified as enhancing sustained engagement with Innowell and creating understanding of DMHTs: the support of a DN with lived experience and collaborative use with clinicians. In this study, incorporating a DN provided young people with both personalized engagement and technological support alongside their regular care teams. The DN offered continuous communication about the rationale and the value of using Innowell through frequent check-ins, engaged in meaningful discussions regarding the young people’s care and health priorities, and subsequently developed tailored, highly-personalized plans for using Innowell in their care. Supporting previous studies that emphasize on client’s understanding on the rationale of DMHTs [51,52] for successful implementation, our findings show that young people’s misunderstandings or concerns about using a DMHT as part of care were alleviated when its purpose and functionality were clearly explained (eg, advising that the technology is not a substitute for in-person clinical care, how to access the login page correctly, and best practices for questionnaire response frequency). Notably, these positive benefits of the DN depended on their ability to build a strong rapport with the participants. These findings suggest that a DN’s experience, empathy, and compassion may be essential characteristics that could determine the effectiveness of the role. Therefore, to ensure scalability and consistency of this role, standardized training protocols should be developed for future DNs, with a particular focus on interpersonal skills, which are crucial for effective client engagement in mental health settings [34].

In addition to the DN’s support, clinicians’ collaborative use of the collected data was essential for fostering young people’s meaningful engagement with the DMHT. Clinician feedback, open communication, and shared decision-making are established motivators for continued engagement and improved treatment outcomes [20,52-54]. Conversely, when data collection is not paired with the use of the data with clinicians, this has been reported to limit clients’ engagement with such DMHTs [25,55].

The findings of this study align with previous research, as clinicians’ use and reviewing of data during sessions emerged as a critical element in helping young people to appreciate the DMHT and its relevance in their care, particularly for contextualizing and monitoring their tracked symptoms. When clinicians did not discuss or review collected symptom data in Innowell during appointments, therefore, reducing the opportunity for collaborative care and communication, this then decreased the perceived clinical relevance of symptom tracking among young people. As a result, this led to disengagement from using the DMHT. It is important to also note that, some factors, such as infrequent appointments (eg, every 3 or 6 months) or discharge from the service, also contributed to reducing the perceived clinical value of Innowell and disengagement from using the technology. However, echoing previous findings, the reluctance of clinicians and services to review progress from routine outcome monitoring data were perceived as a lack of concern for, or indifference to clients’ needs [56,57]. In contrast, collaboratively reviewing longitudinal data enabled proactive responses to young people’s changing needs, such as re-evaluating medication and expediting appointment schedules.

Young people highlight the critical need for collaboration between clinicians and DNs in using DMHTs as part of care. For instance, at baseline visits with the DN, young people were initially enthusiastic about engaging with Innowell to achieve their goals. However, most became more sporadic in their engagement, especially as their clinicians showed reticence to incorporate Innowell into their sessions. The majority of their frustrations came from clinicians not utilizing Innowell effectively or not meeting young people’s expectations for using it. This reiterates established systematic challenges including inadequate training for clinicians and service staff, and lack of supportive environments with clear protocols, strong leadership, and resources such as DNs [57,58]. Further research should focus on extending the DN’s role to support communication between the client-clinician dyad and help clinicians to make effective use of the DMHT to facillitate collaborative care. [32,59].

### Implementation Implication

A plethora of research demonstrates the multifaceted challenges associated with the uptake of DMHTs by mental health clinicians and services. At the clinician level, barriers to implementation include time and resource burdens [60], performance anxiety [61], difficulties integrating with existing electronic systems [60], and insufficient training and leadership support [25,61,62]. At the service level, structural challenges, such as the need for systematic changes and lack of funding, remain significant obstacles to widespread adoption [25,63]. However, the growing evidence of its effectiveness [64-66] and young people’s willingness to adopt this model calls for a concerted effort from all stakeholders to integrate DMHTs effectively into mental health services [67]. Findings from this study indicate that DNs play a key role in alleviating barriers to engagement with DMHTs among young people. Emerging recommendations for DN-driven approaches to address these barriers and support engagement are collated in Textbox 2. Future research should explore longitudinal systematic integration of DMHTs in services. Beyond initial deployment, studies should explore how the adoption of DMHTs can be effectively sustained in services over time. Future research is needed to understand the long-term sustainability of DNs in services and examine how the role can be integrated successfully to reduce the clinical barriers to implementation. Previous research has shown a variety of methods of implementation [31,32], but long-term studies with a DN in a service, measuring their impact, the engagement rate of clinicians, and the outcomes of young people using a DMHT, would significantly improve the current body of research. Of particular note is if there is a “silver bullet” implementation strategy that can be scaled amongst a significant population of services.

**Textbox 2. Recommendations for how digital navigators (DNs) can support engagement with digital mental health tools (DMHTs) in youth mental health.**

1. Provide hands-on onboarding support: assist young people with setting up accounts and completing initial assessments.
2. Explain the purpose and benefits of DMHTs: ensure young people understand how DMHTs can enhance their care (eg, through enhanced communication, progress monitoring, and personalized care coordination).
3. Support symptom tracking integration: help young people to interpret and use their longitudinal data during appointments.
4. Foster ongoing engagement: maintain regular communication with young people (eg, sending reminders for completing questionnaires).
5. Facilitate collaboration for meaningful use: alert clinicians or managers of deteriorations and assist young people’s progress feedback during case review.

### Limitations

Despite the strengths of this study, including longitudinal observation and interviews, the results should be interpreted in consideration of its limitations. First, the study focuses solely on young people’s perspectives, leaving the perspectives of clinicians and services unknown. Examining clinicians’ perspectives may reveal different ways they engage with DMHTs, such as Innowell, that may not be apparent to young people using the DMHT. Second, the study lacks quantitative measurements of young people’s use of Innowell, such as the frequency of questionnaire completion and their symptom trajectories over time. Such metrics could have complemented the qualitative data by characterizing the type of individuals who are more likely to benefit from using DMHTs in their care. Third, qualitative data indicates that the study sample consisted primarily of young people who were familiar with care. While this may limit the generalizability of the findings to those who are new to care, these participants’ previous experiences provided deeper insights into how DMHTs could be better integrated into care. Fourth, the data were collected from young people at a single site (Mind Plasticity), a private mental health service in inner-city Sydney, which likely influenced the socio-economic and cultural profile of participants. This context may not represent the experiences of young people from other diverse backgrounds, those accessing other types of services in different geographical locations, or other age groups with different technology preferences and care needs. As the multi-site clinical trial is ongoing, future analysis of data from other sites will address this limitation by providing insights from more diverse service contexts. Fifth, while the longitudinal design is a strength for this study, the smaller sample size for the follow-up interviews does reduce the impact of these longitudinal findings. The follow-up interviews tended to be opportunistic and are not reflective of dropout of the study itself (beyond the scope of reporting of this study). While the sample size might be limited at follow-up, sufficient information power was reached as no new information was generated between the 6- and 12-month interviews, and so we expect that the 29+ longitudinal interviews provide us with enough detail to understand how views differ over time. Future studies will focus on understanding longitudinal changes in views further by using a more robust sample size. Nevertheless, the data collected from the 6-month interviews had reached sufficient information power and demonstrated young people’s experience of engaging with Innowell as part of usual care. Finally, wider evaluation across multiple clinical settings and contexts would be useful in the future to investigate how different service characteristics and settings shape user experiences, how DN support needs to be adapted for different populations to ensure equity in implementation, and the impact of DNs on implementation of DMHTs across diverse health care environments.

### Conclusion

The implementation of DMHTs has the potential to significantly transform mental health services. Young people recognize the value of using DMHTs in their care, and DNs play an important role in its implementation by reinforcing the rationale behind using the tools and providing engagement and technical support. However, the fidelity of DMHTs and young people’s sustained engagement are contingent on wider contextual factors, including clinician and service engagement. Therefore, it is essential to explore how these factors can be addressed and how DNs can further support services to use DMHTs and long-term engagement.

## Supplementary material

10.2196/70154 Multimedia Appendix 1 Most common questions asked by the digital navigator (DN).

10.2196/70154 Multimedia Appendix 2 Development of themes.

10.2196/70154 Multimedia Appendix 3 Additional quotes for each theme.

## Abbreviations

DMHT: digital mental health tool
DN: digital navigator
MBC: measurement-based care
