# Supplementary material for: Young People’s Experiences Using a Digital Mental Health Tool to Support Their Care in a Real-World Service: Lived Experience–Led Qualitative Study
Source: JMIR Ment Health. 2025 Jun 23;12:e70154. doi: 10.2196/70154 (PMC12208615; doi:10.2196/70154)
Supplement: Multimedia Appendix 1 [file mental-v12-e70154-s001.docx]

**Multimedia Appendix 1**

Most common questions asked by the DN

| Study visit | Question |
| --- | --- |
| Baseline | - What does your current care look like? [I usually know who the treating clinician is]. - [If participant has used Innowell prior to the trial] What’s been your experience of using Innowell as part of your current care?   - From there, I might ask whether their clinician has used it with them - How would you like to use Innowell in your care/with your clinician, what would be meaningful to you? - If you could change anything in your care by using Innowell, what would you like to see change? - Is there something you’d like to get out of this experience in the trial? What’s important to you about being involved in EMPOWERED? - Do you have any feedback or questions - What’s the best way to contact you about Innowell reminders once you’ve been randomised? |
| 3 and 6 months | - What does your current care look now since we last spoke? - What’s been your experience of using Innowell as part of your care during these last few months? Were you able to use it as part of your care with your clinician - Has anything changed for you in the way you use Innowell? [For context, if someone in the intervention group’s Innowell activity increased at the same time as when I ramped up reminders about Innowell]   - Was there something specific that you found helpful about xyz - What would you like, or is there anything that you’d like to see change with how Innowell is being used in your care? - What’s it like having a digital navigator? How do you think other young people who might use Innowell, or a similar digital tool respond to having a digital navigator? - Do you have any feedback or questions - Have contact preferences changed? |
| 12 months | - What does your current care look like? - What’s been your experience of using Innowell this past year?   - Looking back on what some of the key reasons were for you wanting to use it, do you feel like those goals took place? - What’s been most valuable for you about using Innowell, and participating in EMPOWERED and contributing to our research? If anything? - What do you think other young people would find valuable about using Innowell or a similar digital platform as part of their care? - Do you have any questions or feedback for me? |

**NOTES:**

Context

Baseline visit – DN both interviews and onboards to Innowell, discusses Innowell benefits, how to use, integrating into care, use. (during a 1.30 hour baseline visit, interviewed after talking to a research assessor (who takes clinical measures to assess eligibility in trial involvement) which is part of the clinical trial itself)

3 months – 5-min phone call generally. Initiated by the research assessor, followed by a brief interview

6 – in-person or zoom (majority on zoom)

12 - in-person or zoom

Overall – Zoom typically, to facilitate better engagement and scheduling. Consideration for facilitating participation in everyday life.

Innowell use prior/post to interviews – some participant have used Innowell prior to participating and other have not

Considerations for safety, comfort and privacy taken? (e.g., done over the phone)

Outside, public interviews considered but determined to risk harm as it means a clinician is not nearby to assist – decreases safety

In person always at brain and mind centre in the headspace building – increases safety, comfort, privacy, makes clinician support nearby available
