# Supplementary material for: Young People’s Experiences Using a Digital Mental Health Tool to Support Their Care in a Real-World Service: Lived Experience–Led Qualitative Study
Source: JMIR Ment Health. 2025 Jun 23;12:e70154. doi: 10.2196/70154 (PMC12208615; doi:10.2196/70154)

**Multimedia Appendix 2**

**Development of themes**

Initially, two candidate themes were considered, *(De)motivators to use* and *Understanding the user*. However, in discussion, these were found to lack salience as themes. The codes related to these candidate themes were found to be more illustrative of the added role of the DN and, thus, the two candidate themes were reshaped into a new theme (i.e., The digital navigator is more than just a troubleshooter) which incorporated appropriate codes associated with the two withdrawn candidate themes to form two new sub-themes, *Inspiring engagement* and *Creating understanding*. The theme Promoting value for myself and others initially had two sub-themes, however they were not adopted, and the theme is standalone. The theme *Technology as a beneficial tool for communication and collaboration* was renamed to *DMHTs are beneficial for communication and collaboration* to make specific reference to digital mental health tools rather than technology in general.

**Figure S1** Initial and final thematic map, indicating five candidate themes and 10 candidate sub-themes, as well as four finalised themes and six finalised sub-themes. Themes are bolded. Solid, grey boxes represent the finalised themes and sub-themes. Dashed boxes indicate the candidate themes and sub-themes that were not included. Relationship lines between candidate and finalised themes and sub-themes indicate which of the latter integrated codes from the former.


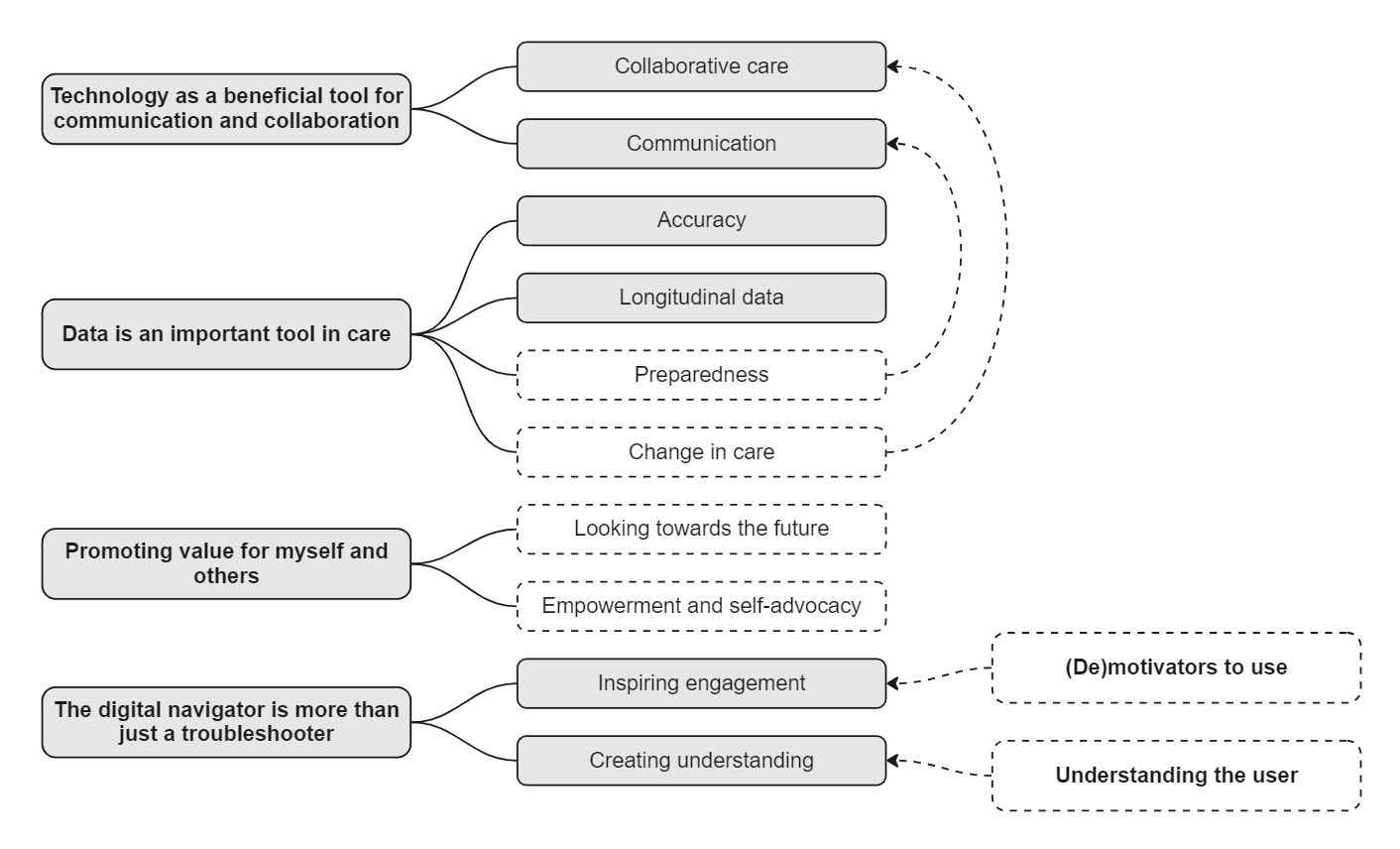

Supplement: Multimedia Appendix 2 [file mental-v12-e70154-s002.docx]
