# Supplementary material for: Young People’s Experiences Using a Digital Mental Health Tool to Support Their Care in a Real-World Service: Lived Experience–Led Qualitative Study
Source: JMIR Ment Health. 2025 Jun 23;12:e70154. doi: 10.2196/70154 (PMC12208615; doi:10.2196/70154)
Supplement: Multimedia Appendix 3 [file mental-v12-e70154-s003.docx]

**Multimedia Appendix 3**

Additional participant quotes from the interviews

| **Themes and subthemes** | **Illustrative quotes** |
| --- | --- |
| 1. **Technology as a beneficial tool for communication and collaboration –**   *Relationships and communication in care and the introduced role of technology* | |
| *1.1 Collaborative Care* | *“What has been the most helpful for me, I was able to bring it up with him and use it as a tool to review my data together…Going into my appointment, I know from Innowell that this is how I’ve been and being able to pull it up and show him, we went into immediate damage control. [My psychiatrist] said straight away ‘Yes, I can see this has been going on for six weeks, this is what we can try, here are some options’, We went straight away into productive mode, exploring those other options during the appointment.” (Participant 1, 3-months visit).*  *“She definitely has been looking at it…when I got bad a couple months ago, [my psychiatrist] sent me a text like 'do I need another appointment sooner'. That was helpful.” (Participant 10, 6-months visit).*  *"He never talked about it [Innowell] but I'm just doing it because of this trial... but it's not that helpful. Talking with a real person, like now, would be inherently better. It's just a website now, so I'm just inputting the data, like on auto-mode". (Participant 3, 6-months visit).*  *“If I turned up at a session and [my clinician] was like 'You didn't use Innowell, we have to do it now to get more out of your appointment', that would be more motivating for me. I think that it really needs to be used with you and your clinician. It has to be seen as valued by your clinician as well.” (Participant 21, 12-months visit).*  *“But for me personally, I do see the benefits of using Innowell and I am willing to make it habitual, and because I know [my psychiatrist] will use it… (Participant 8, Baseline visit).* |
| 1.2 Communication | *"I really enjoy using it. When I'm feeling really low, [Innowell] makes it a lot easier to communicate how I am feeling. It's really helpful for me to tell the truth about my emotions, too…it's easier for me to answer the questionnaires without feeling the guilt and shame to bring it up to [my clinicians]. I don't have to bring it up myself.” (Participant 4, 12-month visit).*  *“I’m a very timid person, even though I’ve been a patient of [my psychiatrist] since 2016, I’m still very much a people pleaser with him. So with Innowell, I was actually able to voice it, and I felt confident to voice it to him because all the information was there. And for me, that’s been what I’ve found most helpful since using Innowell.” (Participant 1, 3-month visit).*  *“It has been useful, it does come up for us [in my appointments]. It's been a point of reference for [my psychiatrist] but also a very good point of reference for myself. It means I can go back and look at my Innowell before I see [my psychiatrist] and remind myself what I wanted to discuss with her.” (Participant 5, 6-months visit).* |
| 1. **Data is an important tool in care –**   *How data is impactful and used by young people and clinicians in care* | |
| 2.1 Accuracy of data for representing mental health conditions | *“When you’re in that mental state and it’s really bad, but you don’t know how long it’s actually been really bad for because it’s all a bit blurry, so when I would’ve reached out to my friends, like ‘hey, when did you start to notice this change in me’, you don’t know for certain how accurate their idea of you might be. And often they would only notice more pronounced changes, which could have happened much later on when I’m already in a really bad state. So Innowell has been really useful because it accurately captures those time points for me, and I found it so interesting because each time I’d update it, I could actually see the consistent declines taking place.” (Participant 1, 3-months visit).*  *“[Without] the different question sets [domains in summary questionnaire], I wouldn't have known that 'oh, this feeling I'm having is because I'm not functioning well at XYZ'. … Innowell has helped me to differentiate those components that make up my mental health and better identify where that underlying feeling I'm having lies, which category it falls into.” (Participant 6, 3-months visit).*  *“The biggest thing for me with the summary questionnaire, some of those questions feel imprecise - it doesn't necessarily capture things as deeply, so I wonder about the accuracy in terms of what the data is looking at.” (Participant 8, 6-months visit).*  *“The questions on possible eating disorders, like 'are you actively doing things' it's a lot more of a complicated issue which doesn't maybe go into enough detail here...” (Participant 5, 6-months visit).* |
| 2.2 Longitudinal data enhancing self-awareness | *“I think Innowell will help me to manage the stress by recognising when things are starting to go down again. It shows you even the slightest of dips on the graph, so I'll be able to see it straight away. And that will help me manage my symptoms by catching it in time.” (Participant 9, 6-months visit).*  *“For me, it's better than journaling. I'm able to learn about myself through the self-reflection. It also gives me the time and space away from everything in therapy, whatever happens in the appointment, it gives me the space to do that self-reflection uninterrupted.” (Participant 22, 6- months visit).*  *I've never had the opportunity to look back and see when things have been good. In my head, I think you focus on the times you've been bad so that becomes all you see in yourself, but it's nice to look at it [the Innowell graphs] and be able to go 'hey, I was pretty good there.” (Participant 10, 6-months visit).*  *“If you have a follow up booked for a month you can still keep engaged in your mental health, and if you eventually have to scale back from having monthly appointments to three appointments a year because of the financial costs involved, if you have access to Innowell it means you don't have to check-out completely. (Participant 6, 6-months visit).* |
| 1. **The impact of the digital navigator in optimising engagement and value -**   *Key roles of the digital navigator in upskilling young people’s understanding of MBC technology and engagement to advance towards personalisation* | |
| 3.1 Inspiring engagement | *"I think the SMSs [Short Message Service messages] that you [the DN] send to me are helpful, especially now that I know you...You want me to do it [Innowell questionnaires] so that makes me want to do it...you’re not going to judge me either way if I do it or not. And you, like, actually care about me using the technology as part of my appointments.” (Participant 7, 6- months visit).*  *“…I think it's a bit of a relief to know that a Digital Navigator is looking at it and monitoring my Innowell when I've updated it…I know it is being looked at, and that gives me reassurance. It's also nice knowing that there's someone there to support you with it if you ever need.”* (*Participant 12*, 6-months visit).  *“[Innowell] only really works for me when it's in person…Every time I've been in the clinic, [the DN] has been really good at motivating me to do it…In-person support [from the DN] is absolutely the way to go.” (Participant 13, 6-months visit)*  *“That’s much better. Now I’ll be able to access it [Innowell] easier. I can see myself doing the questions more consistently now.” (Participant 14, 6-months visit)*  *"In terms of using it [Innowell] in my care at [the service] ...No. Honestly, my appointments are so quick with [my clinician], like 10 minutes or less, so we don't usually go over much or in-depth about anything, really. Which is sad, because I do think it is a useful tool for you to have in your care, but when you consider factors like appointment length ...there's no follow through or indication that your clinician has looked at it or accessed it …” (Participant 23, 6-months visit).*  *"It [Innowell] hasn't really done too much for me to be honest. I don't really see him [clinician] that often, so it doesn't come up.” (Participant 24, 6-months visit).* |
| 3.2 Creating understanding | *“…knowing how it, Innowell, fits into my care, it makes a lot more sense…behind why I should do it and knowing when to do it too.” (Participant 25, Baseline visit).*  *“For me, being able to discuss it with you [the DN] initially and really understand the concepts around using Innowell. Not only did you teach me how to use it, but the ideas you had around how to integrate into my sessions. You really listened to what I wanted to achieve from this experience, and we were able to plan together how I could use Innowell in a way that aligned with my goals for using it in my care.” (Participant 1, 6-months visit).*  “*Explaining the intent of it was really helpful. It [Innowell] was never explained to me how it should be used. It's really helpful to know not just how I'm meant to use it but also knowing how a clinician is supposed to use it. I can see that when your clinician understands this intent too, it has the potential to be really helpful for the person…I can see how Innowell can work when it's used properly. I think I can now say to myself that my previous negative experience of it was because the clinician wasn't using it properly." (Participant 16, Baseline visit).* |
| 1. **Promoting value for myself and others –**   *Meaningful ways technology can be used to add value to oneself and for the broader community via informing service provision change* | |
| *“[my clinician] decides what that [my care] looks like, even though I would like something different. According to him, I’m doing pretty well, but actually, no, I’m really not. But it’s kind of what he decides…I definitely think that the therapeutic relationship hasn't evolved with the needs that I have as I've grown up. I feel like using Innowell can help me to advocate for what I really need, that I can take care of myself and maybe that'll help change things.” (Participant 17, Baseline visit).*  *“I think it is a really good tool. I would like to see it being used more in services. It's very useful all the way round, and I think it's very important that more services use it or a platform like it so young people can have greater access to care.” (Participant 26, 6-months visit).*  *"I'd like the Innowell platform to be seen as part of your care. It needs to be seen as an integral component to how clinicians and a service operate, rather than what they are currently doing. I feel cheated by [my service]...they falsely promote using the platform, they imply that it's been integrated within the service but you can clearly see how tokenistic it is. I'd really like to see the platform be more driven and encouraged by clinicians.” (Participant 19, 6-months visit)*  *"I really believe that this kind of stuff makes things better for people in the future. The emerging young people need this. They need this current young population to be involved in studies like this. I feel like I'm doing my part in the smallest way possible by being involved in the study.” (Participant 1, 6- months visit).* | |
